# Bioclimatic Origin Shapes Phylogenetic Structure of *Tirmania* (Pezizaceae): New Species and New Record from North Africa

**DOI:** 10.3390/jof9050532

**Published:** 2023-04-29

**Authors:** Fatima El-Houaria Zitouni-Haouar, Martin I. Bidartondo, Gabriel Moreno, Juan Ramón Carlavilla, José Luis Manjón, Samir Neggaz, Saida Hanane Zitouni-Nourine

**Affiliations:** 1 Laboratory of Biology of Microorganisms and Biotechnology, Department of Biotechnology, Faculty of Natural and Life Sciences, Oran 1 Ahmed Ben Bella University, Oran 31000, Algeria; samir_neggaz@yahoo.fr; 2 Department of Life Sciences, Imperial College London, Silwood Park, Ascot, London SL5 7PY, UK; m.bidartondo@imperial.ac.uk; 3 Ecosystem Stewardship, Royal Botanic Gardens, Kew, Richmond TW9 3DS, UK; 4 Departamento de Ciencias de la Vida, Facultad de Biología, Universidad de Alcalá, 28805 Alcalá de Henares, Madrid, Spain; gabriel.moreno@uah.es (G.M.); juan.carlavilla@gmail.com (J.R.C.); josel.manjon@uah.es (J.L.M.); 5 Pharmaceutical Development Research Laboratory, Department of Pharmacy, Faculty of Medicine, Oran 1 Ahmed Ben Bella University, Oran 31000, Algeria; zitounihanane83@gmail.com

**Keywords:** Ascomycota, desert truffles, systematics, new species, speciation, bioclimate

## Abstract

The phylogenetic relationships among *Tirmania* were investigated using the internal transcribed spacer (ITS) and large subunit (LSU) regions of the nuclear-encoded ribosomal DNA (rDNA) and compared with morphological and bioclimatic data. The combined analyses of forty-one *Tirmania* samples from Algeria and Spain supported four lineages corresponding to four morphological species. Besides the two previously described taxa, *Tirmania pinoyi* and *Tirmania nivea*, here we describe and illustrate a new species, *Tirmania sahariensis* sp. nov., which differs from all other *Tirmania* by its distinct phylogenetic position and its specific combination of morphological features. We also present a first record of *Tirmania honrubiae* from North Africa (Algeria). Our findings suggest that restrictions imposed by the bioclimatic niche have played a key role in driving the speciation process of *Tirmania* along the Mediterranean and Middle East.

## 1. Introduction

*Tirmania* Chatin is an edible hypogeous desert truffle mostly endemic to arid and desertic areas of the Mediterranean region and the Middle East. Species of this genus have long been appreciated as a popular delicacy of the Arabian diet and were frequently used in the ethnomedicine of the North African Bedouins to treat several ailments. Moreover, in some areas of the Arabian Gulf region, *Tirmania* was considered as an extremely prestigious food item. Indeed, Gulf royal families claimed the truffle crop during seasons of abundance and had truffle lands patrolled until most of the crop was harvested [1]. Similarly to other mycorrhizal desert truffles, *Tirmania* species form a symbiotic mycorrhizal relationship with the roots of exclusive host plants. Members of the Cistaceae family and most notably annual and perennial species of the *Helianthemum* genus have been identified as the most common host plants preferred by the two desert truffles sister species *Tirmania* and *Terfezia* [1,2,3,4,5,6]). These host plants species were the main key behind the domestication achievement of desert truffles as a niche crop. In fact, successful desert truffle plantations led to the cultivation of two *Terfezia* species, *Terfezia claveryi* and *Terfezia boudieri*, mycorrhizing two species of *Helianthemum*, *Helianthemum almeriense* and *Helianthemum sessiliflorum*, respectively. Good desert truffle yields were obtained with these *Helianthemum* mycorrhized seedlings prepared in a nursery and planted afterwards in appropriate cultivation plots with effective irrigation regimes [7].

The genus *Tirmania* was raised by Chatin [8,9] to accommodate desert truffle specimens received from Algeria (North Africa) displaying at that time a specific combination of morphological criteria characterized mostly by a white peridium and smooth, ellipsoid spores. The etymology of the generic name *Tirmania* came from *M. Tirman*, the general governor of Algeria who sent the type specimens to Chatin, while the type species of the genus earned the epithet of *Tirmania africana* in reference to its origin [8]. Two *Tirmania* species *Tirmania cambonii* and *Tirmania ovalispora* were proposed subsequently by Chatin and Patouillard, respectively [8,10]. However, the work of Trappe [11] synonymized the *Tirmania* species described previously and proposed the new combination *Tirmania nivea* to represent the *Tirmania* taxon with broadly ellipsoid, smooth spores. He further offered a new effective taxonomic identification tool allowing the distinction of *Tirmania* from its closest taxa based on the amyloid reaction (green to blue color) of its asci in response to Melzer’s solution. This specific amyloid reaction allowed the transfer of *Tirmania* from the Terfeziaceae [11] to the Pezizaceae family [12]. Molecular phylogenetic studies that have been undertaken later on the Pezizaceae clearly confirmed and demonstrated that *Tirmania* belongs to this family [3,13,14,15,16,17,18]. On the other hand, Maire [19] described a new *Tirmania* species with globose minutely roughened spores which he affiliated to the *Terfezia* group under the name *Terfezia pinoyi* despite noting great similarities between the described specimen and the type species of *Tirmania*. This new species was transferred many years later to *Tirmania* by Malençon [20]. Alsheikh and Trappe [1] published a global taxonomic monograph of the genus *Tirmania*. They studied morphological and anatomical features of many fresh ascomata and several valuable herbarium specimens of *Tirmania* and concluded that all the different *Tirmania* taxa described previously were erected based on modest morphological differences and should be represented under two species namely *Tirmania nivea* and *Tirmania pinoyi*.

*Tirmania* was traditionally restricted to North Africa and Western Asia [1,11]) until the first record of this genus was reported from the Tabernas Desert (Almería, Southern Spain) of the Iberian Peninsula [21]. Three years later, Moreno et al. [22] re-examined the material studied by Moreno-Arroyo et al. [21] at the the Real Jardín Botánico of Madrid. The collection was originally described and recorded as *T*. *pinoyi*; however, Moreno et al. [22] emended the taxonomic misidentification at the species level leading to the abolishment of *T. pinoyi* from the Spanish hypogeous mycoflora and, consequently, amended the European Catalogue of hypogeous fungi by the introduction of a first new *T. nivea* record.

Recent molecular taxonomic revisions on the genus *Terfezia* have proved that this taxon is the most species-rich among the desert truffles. Taxonomic revisions on this genus are still ongoing and discoveries of new species considerably increased the richness of *Terfezia* over the Mediterranean and the Middle Eastern region [5,23,24,25,26,27,28,29,30,31]. However, the sister genus *Tirmania* has always been regarded as a genetically conserved, less diverse taxon. Nonetheless, a third *Tirmania* species *Tirmania honrubiae* sp. nov. was recently described from the Middle East (United Arab Emirates) [32]. The main goal of the present study was to investigate the diversity of the genus *Tirmania* in North Africa with a more extensive sampling from Algeria. Novel *Tirmania* species and a new record are herein described from North Africa by means of morphological and molecular phylogenetic investigations of rDNA sequences. Target genes were also sequenced for the herbarium specimen of the first *T. nivea* record in Spain. In addition, we provide an updated taxonomic key to species of *Tirmania* revised so far in light of new molecular and morphological data. Ecological factors especially climatic zones of the *Tirmania* DNA sequences representing all the major clades of the genus along its geographic distribution area in the Mediterranean and Middle Eastern countries were determined according to the Köppen–Geiger global climate classification to provide new insights into the factors driving speciation and evolutionary phenomena in *Tirmania*.

## 2. Materials and Methods

### 2.1. Field-Collected Fungal Specimens

**Figure 1:**
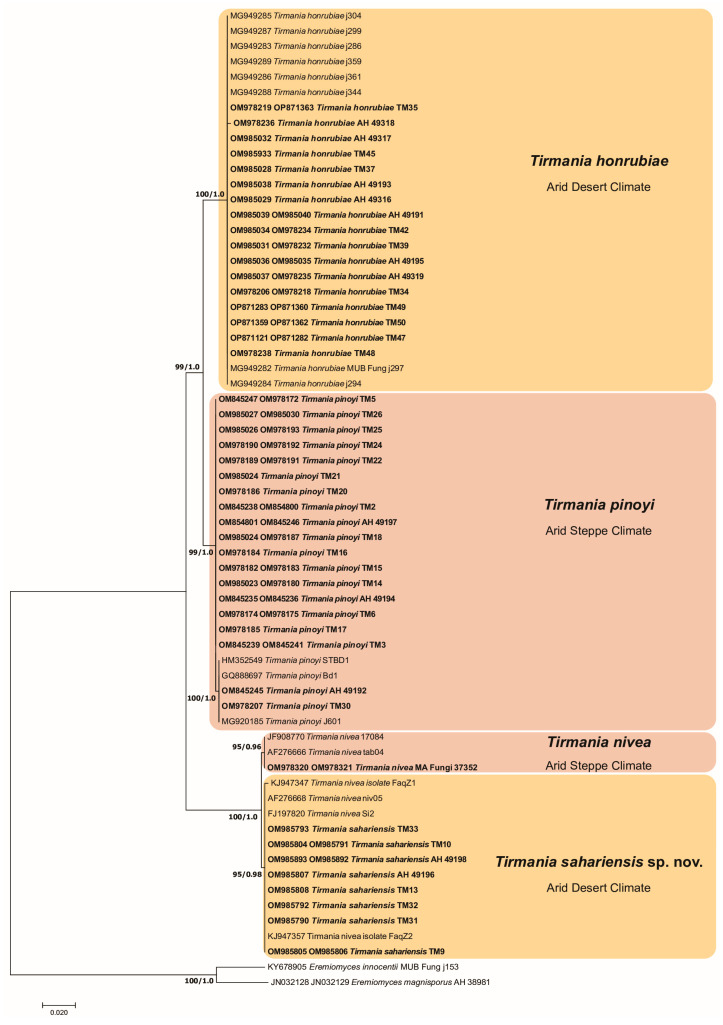
Maximum likelihood (ML) phylogram of *Tirmania* species inferred from the concatenated DNA sequence data of ITS and 28S rDNA gene regions with *Eremiomyces innocentii* and *Eremiomyces magnisporus* as outgroups. RAxML bootstrap support (BS) values equal to or above 70% and Bayesian posterior probability (PP) scores equal to or greater than 0.95 are shown at the nodes. The sequences obtained in the present study are highlighted in bold. Bar = 2 changes/100 characters.

Specimens of *Tirmania*, commonly called white desert truffles, were collected from the Western steppe and desert regions of Algeria which are characterized by a cold/hot arid steppe climate and cold/hot arid desert climate, respectively (Table 1; Figure 1), according to the Köppen–Geiger global climate classification [33]. The locations of desert truffles ascocarps were detected by cracked humps on the soil surface formed by the swelling of the fruitbodies beneath the ground. The typical ascomata cracks were often found near to the common natural host plants of desert truffles, *Helianthemum* spp., or to some other xerophilous plants. Ascocarps were extracted from the cracks using wooden or metal digging sticks. *Tirmania* specimens were primarily selected among the harvested desert truffles based on their whitish peridium. The presumed *Tirmania* ascomata were then sun-dried on a sieve and stored in sealed paper bags, labelled with collection details and macromorphological characteristics, pending further morphological and genetic identification. The Spanish field-collected *Tirmania* specimen MA fungi 37352, harvested from the Tabernas Desert (Almería, Southern Spain) and housed in the herbarium of the Real Jardín Botánico of Madrid, was also studied and used as a reference material from the Iberian Peninsula. Several dried samples representing the three major genetic clades of *Tirmania* encountered in Algeria, including the new species and the new record, were deposited in the Herbarium AH of the University of Alcalá de Henares, Spain, under voucher specimen numbers listed in Table 1.

### 2.2. Morphological Characterization

Macromorphological features including color, structure, shape, and size of ascomata (peridium and gleba) were described from fresh specimens. Micromorphology of the inner and outer structure of the peridium was examined and recorded from rehydrated hand-sectioned dried ascocarp tissues. Spore and asci shape, color, and number of ascospores per ascus were evaluated in distilled water, 5% KOH, and cotton blue-lactophenol. Small samples of gently squashed gleba were observed in Melzer’s reagent [34] to assess the amyloid reaction of asci and spore walls. Spore and asci dimensions were determined from gleba squash preparations in distilled water mounts on at least 50 mature spores and asci with the aid of an Olympus CX22 light microscope equipped with an ocular micrometer. For scanning electron microscopy (SEM) observations, samples were firstly prepared according to the critical point drying technique prior to mounting, following Moreno et al. [35]. Ascospore ornamentation was examined and photographed using the scanning electron microscopy (SEM) Zeiss DSM-950 instrument at the University of Alcalá (Spain).

**Table 1:** Geographic origin, Ecological data, and GenBank accession IDs of the newly sequenced *Tirmania* collections and reference sequences used in the molecular phylogenetic study. **BSK**: Cold arid steppe climate; **BSH**: Hot arid steppe climate; **BWK**: Cold arid desert climate; **BWH:** Hot arid desert climate. N/F = no details found. N/A: no data available.

Species	Voucher Specimen	Country of Origin	Locality	Host Plant	Bioclimatic Zone (Köppen–Geiger Climate Classification)	GenBank Accession IDs	
						ITS	28S LSU
***Tirmania pinoyi* lineage**							
***Tirmania pinoyi***	AH 49194	Algeria	Naama, Mecheria, El Biodh	*Helianthemum* sp.	BSK	OM845235	OM845236
***Tirmania pinoyi***	TM2	Algeria	Naama, Mecheria, El Biodh	*Helianthemum* sp.	BSK	OM845238	OM854800
***Tirmania pinoyi***	TM3	Algeria	Naama, Mecheria, El Biodh	*Helianthemum* sp.	BSK	OM845239	OM845241
***Tirmania pinoyi***	AH 49192	Algeria	Naama, Mecheria, Bougarne	*Helianthemum* sp.	BSK	OM845245	N/A
***Tirmania pinoyi***	TM5	Algeria	Naama, Mecheria, Bougarne	*Helianthemum* sp.	BSK	OM845247	OM978172
***Tirmania pinoyi***	TM6	Algeria	Naama, Mecheria, Bougarne	*Helianthemum* sp.	BSK	OM978174	OM978175
***Tirmania pinoyi***	TM14	Algeria	Tiaret, Ksar Chellala, Zmalet El-Amir Abdelkader, Bouchouat	*Helianthemum hirtum, Helianthemum salicifolium*	BSK	OM985023	OM978180
***Tirmania pinoyi***	TM15	Algeria	Tiaret, Ksar Chellala, Zmalet El-Amir Abdelkader, Bouchouat	*Helianthemum hirtum, Helianthemum salicifolium*	BSK	OM978182	OM978183
***Tirmania pinoyi***	TM16	Algeria	Tiaret, Ksar Chellala, Zmalet El-Amir Abdelkader, Bouchouat	*Helianthemum hirtum, Helianthemum salicifolium*	BSK	OM978184	N/A
***Tirmania pinoyi***	TM17	Algeria	Tiaret, Ksar Chellala, Zmalet El-Amir Abdelkader, Bouchouat	*Helianthemum hirtum, Helianthemum salicifolium*	BSK	OM978185	N/A
***Tirmania pinoyi***	TM18	Algeria	Tiaret, Ksar Chellala, Zmalet El-Amir Abdelkader, Bouchouat	*Helianthemum hirtum* *Helianthemum salicifolium*	BSK	OM985024	OM978187
***Tirmania pinoyi***	AH 49197	Algeria	Tiaret, Ksar Chellala, Zmalet El-Amir Abdelkader, Bouchouat	*Helianthemum hirtum* *Helianthemum salicifolium*	BSK	OM854801	OM845246
***Tirmania pinoyi***	TM20	Algeria	Tiaret, Hamadia, Rechaiga, Benhamed	*Helianthemum hirtum*	BSK	OM978186	N/A
***Tirmania pinoyi***	TM21	Algeria	Tiaret, Hamadia, Rechaiga, Benhamed	*Helianthemum hirtum*	BSK	OM985024	N/A
***Tirmania pinoyi***	TM22	Algeria	Tiaret, Hamadia, Rechaiga, Benhamed	*Helianthemum hirtum*	BSK	OM978189	OM978191
***Tirmania pinoyi***	TM24	Algeria	Tiaret, Hamadia, Rechaiga, Benhamed	*Helianthemum hirtum*	BSK	OM978190	OM978192
***Tirmania pinoyi***	TM25	Algeria	Tiaret, Hamadia, Rechaiga, Benhamed	*Helianthemum hirtum*	BSK	OM985026	OM978193
***Tirmania pinoyi***	TM26	Algeria	Tiaret, Hamadia, Rechaiga, Benhamed	*Helianthemum hirtum*	BSK	OM985027	OM985030
***Tirmania pinoyi***	TM30	Algeria	Tiaret, Hamadia, Rechaiga, Benhamed	*Helianthemum hirtum*	BSK	OM978207	N/A
***Tirmania pinoyi***	Bd1	Iran	Hormozgan, Bishederaz	*Helianthemum salicifolium*	BSK	GQ888697 *	N/A
***Tirmania pinoyi***	j601	Syria	Damasco	N/F	BSK	MG920185 *	N/A
***Tirmania pinoyi***	STBD1	Iran	Hormozgan, Bishederaz	*Helianthemum salicifolium*	BSK	HM352549 *	N/A
***Tirmania honrubiae* lineage**							
***Tirmania honrubiae***	TM34	Algeria	Béchar, Béni Ounif, Oued Namous	*Helianthemum lippii*	BWH	OM978206	OM978218
***Tirmania honrubiae***	TM35	Algeria	Béchar, Béni Ounif, Oued Namous	*Helianthemum lippii*	BWH	OM978219	OP871363
***Tirmania honrubiae***	AH 49191	Algeria	Béchar, Béni Ounif, Oued Namous	*Helianthemum lippii*	BWH	OM985039	OM985040
***Tirmania honrubiae***	TM37	Algeria	Béchar, Abadla, Hammaguir	*Helianthemum lippii*	BWH	OM985028	N/A
***Tirmania honrubiae***	AH 49316	Algeria	Béchar, Abadla, Hammaguir	*Helianthemum lippii*	BWH	OM985029	N/A
***Tirmania honrubiae***	TM39	Algeria	Béchar, Abadla, Hammaguir	*Helianthemum lippii*	BWH	OM985031	OM978232
***Tirmania honrubiae***	AH 49195	Algeria	Béchar, Abadla, Hammaguir	*Helianthemum lippii*	BWH	OM985036	OM985035
***Tirmania honrubiae***	AH 49317	Algeria	Béchar, Abadla, Hammaguir	*Helianthemum lippii*	BWH	OM985032	N/A
***Tirmania honrubiae***	TM42	Algeria	Béchar, Abadla, Oglat Braber	*Helianthemum lippii*	BWH	OM985034	OM978234
***Tirmania honrubiae***	AH 49319	Algeria	Béchar, Abadla, Oglat Braber	*Helianthemum lippii*	BWH	OM985037	OM978235
***Tirmania honrubiae***	AH 49193	Algeria	Béchar, Abadla, Oglat Braber	*Helianthemum lippii*	BWH	OM985038	N/A
***Tirmania honrubiae***	TM45	Algeria	Béchar, Abadla, Oglat Braber	*Helianthemum lippii*	BWH	OM985033	N/A
***Tirmania honrubiae***	AH 49318	Algeria	Béchar, Abadla, Oglat Braber	*Helianthemum lippii*	BWH	OM978236	N/A
***Tirmania honrubiae***	TM47	Algeria	Béni Abbès, Boulaadam	*Helianthemum lippii*	BWH	OP871121	OP871282
***Tirmania honrubiae***	TM48	Algeria	Béni Abbès, Boulaadam	*Helianthemum lippii*	BWH	OM978238	N/A
***Tirmania honrubiae***	TM49	Algeria	Laghouat, Hassi R’Mel	*Helianthemum* sp.	BWH	OP871283	OP871360
***Tirmania honrubiae***	TM50	Algeria	Laghouat, Hassi R’Mel	*Helianthemum* sp.	BWH	OP871359	OP871362
***Tirmania honrubiae***	j286	United Arab Emirates	Abu Dhabi, Ghantoot	*Helianthemum lippii*	BWH	MG949283 *	N/A
***Tirmania honrubiae***	j294	United Arab Emirates	Abu Dhabi, Ghantoot	*Helianthemum lippii*	BWH	MG949284 *	N/A
***Tirmania honrubiae***	j297	United Arab Emirates	Abu Dhabi, Ghantoot	*Helianthemum lippii*	BWH	MG949282 *	N/A
***Tirmania honrubiae***	j299	United Arab Emirates	Abu Dhabi, Ghantoot	*Helianthemum lippii*	BWH	MG949287 *	N/A
***Tirmania honrubiae***	j304	United Arab Emirates	Abu Dhabi, Ghantoot	*Helianthemum lippii*	BWH	MG949285 *	N/A
***Tirmania honrubiae***	j344	United Arab Emirates	Abu Dhabi, Seih Sadira	*Helianthemum lippii*	BWH	MG949288 *	N/A
***Tirmania honrubiae***	j359	United Arab Emirates	Abu Dhabi, Ghantoot	*Helianthemum lippii*	BWH	MG949289 *	N/A
***Tirmania honrubiae***	j361	United Arab Emirates	Abu Dhabi, Ghantoot	*Helianthemum lippii*	BWH	MG949286 *	N/A
***Tirmania nivea* lineage**							
***Tirmania nivea***	MA Fungi 37352	Spain	Almeria, Tabernas Desert	Probably *Helianthemum almeriense*	BSK	OM978320	OM978321
***Tirmania nivea***	tab04	Spain	Almeria, Tabernas Desert	N/F	BSK	AF276666 *	N/A
***Tirmania nivea***	17084	Israel	Ze’elim	N/F	BSH	JF908770 *	N/A
***Tirmania sahariensis* sp. nov. lineage**							
***Tirmania sahariensis***	TM9	Algeria	Naama, Ain Sefra	*Helianthemum lippii*	BWK	OM985805	OM985806
***Tirmania sahariensis***	TM10	Algeria	Naama, Ain Sefra	*Helianthemum lippii*	BWK	OM985804	OM985791
***Tirmania sahariensis***	AH 49198	Algeria	Naama, Ain Sefra	*Helianthemum lippii*	BWK	OM985893	OM985892
***Tirmania sahariensis***	AH 49196	Algeria	Naama, Ain Sefra	*Helianthemum lippii*	BWK	OM985807	N/A
***Tirmania sahariensis***	TM13	Algeria	Naama, Ain Sefra	*Helianthemum lippii*	BWK	OM985808	N/A
***Tirmania sahariensis***	TM31	Algeria	Béni Abbès, Tabelbala, Oued Daoura	*Helianthemum lippii*	BWH	OM985790	N/A
***Tirmania sahariensis***	TM32	Algeria	Béni Abbès, Tabelbala, Oued Daoura	*Helianthemum lippii*	BWH	OM985792	N/A
***Tirmania sahariensis***	TM33	Algeria	Béchar, Abadla, Hammaguir, Erg Serhen	*Helianthemum lippii*	BWH	OM985793	N/A
***Tirmania nivea***	FaqZ1	Qatar	N/F	N/F	BWH	KJ947347 *	N/A
***Tirmania nivea***	FaqZ2	Qatar	N/F	N/F	BWH	KJ947357 *	N/A
***Tirmania nivea***	niv05	Kuwait	N/F	*Helianthemum salicifolium*	BWH	AF276668 *	N/A
***Tirmania nivea***	Si2	Iran	Kerman, Baghaat, Sirjan	*Helianthemum salicifolium*	BWK	FJ197820 *	N/A

* Sequences retrieved from GenBank. The others are new sequences obtained in the present study.

### 2.3. Genomic DNA Extraction, PCR Amplification, and Sequencing of Ribosomal DNA

The majority of the Algerian *Tirmania* samples (93%) were genetically analyzed at the Jodrell laboratory of the Royal Botanic Gardens (Kew, London, UK). Genomic DNA was extracted from dried glebal tissues following the CTAB method of Gardes and Bruns [36] with DNA binding and purification according to Bidartondo et al. [37]. After rigorous disinfection of the peridium, 20 mg of clean glebal tissues were removed from the inner part of ascomata to be suspended in 300 µL of cetyltrimethyl ammonium bromide (CTAB) lysis buffer (100-mM Tris-HC1 (pH 8.0), 1.4 M NaCl, 20 mM EDTA, 2% CTAB, 1% PVP-40). The buffer-suspended samples were frozen at −20 °C for 30 min and then thawed in a 45 °C heat block. The glebal tissues were crushed afterwards with a micropestle and incubated at 65 °C for 30 min; then, 300 µL of chloroform was added to each sample and mixed twice by pulse vortexing for 10 sec. Following centrifugation at 13,200 rpm for 15 min, the upper phase (~200 µL) was removed from each tube and transferred to a new centrifuge tube. The DNA was then purified using a GeneClean III kit (Qbiogene, Carlsbad, CA, USA) as described by the manufacturer. Polymerase chain reaction (PCR) amplifications were performed on two target ribosomal genes (ITS and LSU) generally considered as fungal DNA barcoding markers. The internal transcribed spacer (ITS) and 28S large subunit (LSU) regions of nuclear-encoded ribosomal DNA (rDNA) were amplified using the fungal-specific primers ITS1-F/ITS4 [36,38]) and the primers pair LR0R-LR5 [39,40], respectively. Aliquots of 2.5 μL of DNA templates from the *Tirmania* samples were combined with 7.5 μL of 2× PicoMaxx^®^ high-fidelity PCR Master mix (Stratagene, CedarCreek, TX, USA). The PCR cycling was carried out on an Eppendorf Mastercycler Pro^®^ thermocycler equipped with a vapo protect lid (Eppendorf, Hamburg, Germany). Amplifications were performed with an initial denaturation step at 95 °C for 2 min, followed by 32 cycles of denaturation at 95 °C for 40 s, annealing at 53 °C for 30 s and extension at 72 °C for 3 min 50 s, with a final extension step of 72 °C for 5 min. The PCR amplicons were confirmed by checking with ethidium-bromide-stained agarose gels (1.5%) electrophoresed in 0.5× TBE buffer at 80 V for 25 min and visualized using a UV-light imaging unit UVP GelStudio (Analytik Jena; Upland, CA, USA). Positive PCR products were then purified with exonuclease I and shrimp alkaline phosphatase ExoSAP-IT (USB/GE Healthcare, Buckinghamshire, UK) following the manufacturer’s instructions. The DNA sequencing was performed on an ABI 3730 Genetic Analyzer (Applied Biosystems, Foster City, CA, USA) using a BigDye v.3.1 Cycle Sequencing Kit (Applied Biosystems, Foster City, CA, USA) following purification by ethanol and EDTA precipitation. The DNA extraction, PCR amplifications and ITS-LSU rDNA sequencing of the 7% remaining Algerian *Tirmania* samples investigated in the present work were performed at the University of Alcalá de Henares (Spain) as described by Zitouni-Haouar et al. [5,41]. The molecular characterization of the Spanish *Tirmania* sample MA Fungi 37352 was also conducted at the University of Alcalá de Henares. The DNA extraction was performed on a dry specimen, employing a modified protocol based on Murray and Thompson [42]. PCR amplifications targeting the ITS and LSU rDNA sequences were conducted according to Mullis and Faloona [43]. Amplification reactions included 35 cycles with an annealing temperature of 54 °C. The PCR products were checked using 1% agarose gel electrophoresis. Positive amplicons were purified and sequenced with one or both PCR primers at the ALVALAB (Oviedo, Spain). All the ITS and LSUgenerated sequences have been submitted to GenBank under the accession numbers reported in Table 1.

### 2.4. Molecular Phylogeny Construction

Preliminary taxonomic identification of the ITS and 28S rDNA sequences generated in this study was achieved by conducting a similarity search using the BLAST algorithm [44] of GenBank (http://www.ncbi.nlm.nih.gov/blast (accessed on 5 January 2022)). Each electrophoretogram was checked visually and its corresponding sequence was edited manually using Chromas software version 2.1.10 in order to remove low-quality peaks. DNA sequences obtained in the present work were then aligned to those most similar in a single combined ITS and 28S rDNA alignment using the MUSCLE algorithm [45] implemented in MEGA 11.0. software [46]. The closest reference sequences were selected to represent the three currently accepted taxa in *Tirmania* from Díez et al. [3], Jamali and Banihashemi [47], Osmundson et al. [48], and Morte et al. [32]. Sequences from *Eremiomyces innocentii* and *Eremiomyces magnisporus* were chosen for outgroup comparison. Maximum likelihood phylogenies and Bayesian analyses were performed on the final alignment to infer phylogenetic relationships among the *Tirmania* group. Analyses of the best-scoring maximum likelihood tree were conducted in RAxML-HPC2 on XSEDE [49] using 1.000 bootstrap replications. Evolutionary models were determined using jModelTest [50] with GTR + G for ITS and GTR + G + I for LSU being selected as the best models. Bayesian phylogenetic inference was carried out using MrBayes 3.2.6 [51]. Four simultaneous independent chains were run from random trees for 10.000.000 generations. Trees were sampled every 1000th generation, and the first 25% of the sampled trees from each run were discarded as burn-in. Only significant branch support is displayed at the nodes with a maximum likelihood bootstrap support (MLB) ≥ 70% and Bayesian posterior probability (PP) ≥ 0.95.

## 3. Results

### 3.1. Phylogenetic Analysis

The combined phylogenetic tree accommodated 89 sequences listed in Table 1. The ITS and LSU amplicons size of the 45 genotyped *Tirmania* ascomata were 602 and 634 bp in the final alignment, with 217 and 63 variable positions, respectively. The maximum likelihood and Bayesian inference trees yielded similar topologies for both ITS and 28S rDNA gene regions, and the concatenated data set identified the same supported clades. Phylogenetic analysis of ITS and LSU sequence data from the *Tirmania* specimens investigated in this study in relationship with the *Tirmania* reference sequences retrieved from GenBank revealed two major lineages highly supported by bootstrap and posterior probabilities values (BS: 99%, PP: 1.00; BS: 100%, PP: 1.00). The two major lineages separated into four strongly supported and well-defined clades corresponding to four morphologically distinct *Tirmania* species. Moreover, each genetic clade identified in *Tirmania* ITS + LSU phylogenies correlated well with a specific bioclimatic pattern. Two of the four clades represented two *Tirmania* taxa previously reported from the Mediterranean region and the Middle East, namely *T. pinoyi* and *T. nivea*. Several rDNA sequences from Algerian samples studied in the present work formed a robustly supported clade (BS: 100%, PP: 1.00) with *T. honrubiae* sequences retrieved from GenBank supporting their affiliation to this taxon and representing, therefore, a first record of this species from North Africa. The remaining fourth clade was formed by *Tirmania* samples showing specific morphological characters not encountered even in their genetically closest sister species *T. nivea*, thus, evidencing their distinct taxonomic status. We, therefore, propose a novel taxonomic combination to accommodate samples of the fourth *Tirmania* clade. The designation of *Tirmania sahariensis* as a new species is supported by ITS/LSU rDNA analyses and morphological features (Figure 1).

### 3.2. Taxonomy

***Tirmania sahariensis*** Zitouni-Haouar, Bidartondo, G. Moreno, Carlavilla & Manjón sp. nov.

MycoBank MB847872

GenBank: OM985893, OM985892 (Holotype).

Figure 2

**Type.** ALGERIA, Naama, Ain Sefra, under *Helianthemum lippii* (L.) Dum. Cours., hypogeous, mostly solitary, 12 April 2012, F.E.-H. Zitouni-Haouar (holotype: AH49198, paratype: AH49196).

**Diagnosis.***Tirmania sahariensis* differs from its sisters *Tirmania* species (*T. nivea, T. pinoyi* and *T. honrubiae*) by having a combination of smooth globose and ellipsoid spores compared to its closest phylogenetically related species *T. nivea* which is characterized by strictly ellipsoid smooth spores [22]. This species is also recognized by its unusual maximum number of spores per ascus which can reach 10 spores. The distinct phylogenetic position of *T. sahariensis* separates it furthermore from the rest of *Tirmania* species. *T. honrubiae* is characterized by globose spores ornamented by low-rounded warts. *T. pinoyi* has spherical minutely reticulate spores.

**Etymology.***“sahariensis”* epithet in reference to its Saharan habitat.

**Figure 2:**
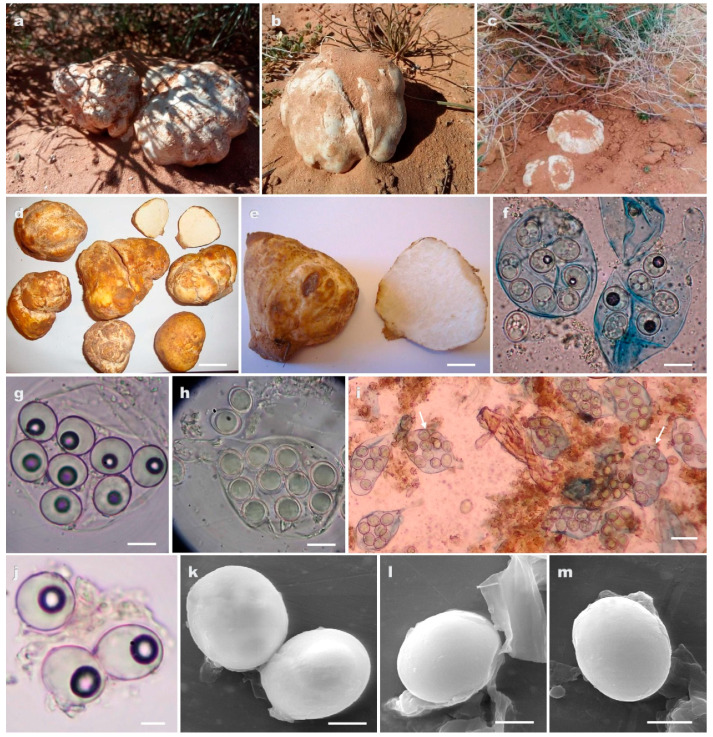
(**a**–**m**) *Tirmania sahariensis* sp. nov.: (**a**–**c**) ascomata *in situ*, under their host plant *Helianthemum lippii*; (**c**,**d**) ascomata showing peridial surface; (**e**) gleba in cross section; (**f**) light microscopy of asci harboring a mixture of ellipsoid and globoses spores in Melzer’s reagent; (**g**) ascus with exclusively ellipsoid spores; (**h**,**i**) asci containing ten spores (arrows); (**j**) ellipsoid and globoses ascospores; (**k**–**m**) scanning electron microscopy of ascospores. Scale bars: 3.3 cm (**d**), 2.1 cm (**e**), 15 μm (**f**,**h**), 10 μm (**g**), 30 μm (**i**), and 5 μm (**j**–**m**).

**Description. *Ascomata*** hypogeous to partially or occasionally completely emergent at maturity; subglobose to ellipsoid, sometimes lobed or turbinate with small basal attachment, smooth or covered with shallow crevices; 2.2–7.4 × 3.3–9.4 cm in size, 7–125 g fresh weight; off white or white yellowish when young, becoming yellowish brown to orange brown with age or after desiccation (Figure 2a–d). *Gleba* solid, fleshy, with subglobose to elongate pale yellow to light pink pockets of fertile tissue, separated by whitish sterile veins (Figure 2e). *Odor* and *taste* very pleasant.

***Peridium*** ± 200 µm thick, consisting of two layers. The outer layer is composed of appressed, interwoven hyphae, 5–11 μm in diameter at septa, with inflated pale yellow or hyaline scattered outermost cells, walls 0.5–1 μm thick. The inner peridium differentiated as hyaline, interwoven septate hyphae (textura intricata), 13–30 μm broad with innermost cells subglobose, inflated to 20–42 μm diam, thin-walled and hyaline. ***Asci*** amyloid; mostly ellipsoid to pyriform or subglobose; 50–96 × 39–55 μm, with short stipitate (4–) 7–15 × 8–27 (–37) μm; walls 0.5–1.5 μm thick, harboring 6–(8–10) spores either exclusively ellipsoid or mixed with 1 to 3 globose spores (Figure 2f–i). ***Ascospores*** ellipsoid to subglobose, (10–) 11–14 × 12.5–18.5 (–19) μm, or globose, (10.5–) 12 × 15 μm diam.; hyaline with numerous small lipid guttules in immature spores merging into a single large guttule at maturity with a de Bary bubble (in Melzer’s reagent); the walls are 1–1.5 μm thick and two-layered: outer layer smooth, inner layer smooth to occasionally very minutely roughened with irregularly dispersed small ridges (up to 0.4 μm high) (Figure 2f–m). 

**Distribution and Ecology.** ALGERIA, Naama, Ain Sefra; Béchar, Abadla, Hammaguir, Erg Serhen; Béni Abbès, Tabelbala, Oued Daoura. Hypogeous, solitary or gregarious (two ascomata), in sandy alkaline soil associated with *Helianthemum lippii.* This species is locally called «*terfess labiadh*» which means white truffle. Ascocarps are collected from the beginning of December to the end of May.

**Additional collections examined.** ALGERIA, Naama, Ain Sefra, April 2012, leg. F.E.-H. Zitouni-Haouar, paratype AH49196 (ITS sequence GenBank OM985807), TM9 (ITS sequence GenBank OM985805, LSU sequence GenBank OM985806), TM10 (ITS sequence GenBank OM985804, LSU sequence GenBank OM985791), TM13 (ITS sequence GenBank OM985808); ALGERIA, Béni Abbès, Tabelbala, Oued Daoura, April 2012, leg. F.E.-H. Zitouni-Haouar, TM31 (ITS sequence GenBank OM985790), TM32 (ITS sequence GenBank OM985792); ALGERIA, Béchar, Abadla, Hammaguir, Erg Serhen, March 2015, leg. F.E.-H. Zitouni-Haouar, TM33 (ITS sequence GenBank OM985793).

***Tirmania nivea*** (Desf.: Fr.) Trappe.

**Descriptions.** See Moreno et al. [22]

**Collections examined.** SPAIN, Almería, Tabernas Desert, in sandy soils, 29 May 1995, leg. P. Rodriguez, MA-Fungi 37,352 (ITS sequence GenBank OM978320, LSU sequence GenBank OM978321).

***Tirmania honrubiae*** Morte, Bordallo & Ant. Rodr.

Figure 3

**Figure 3:**
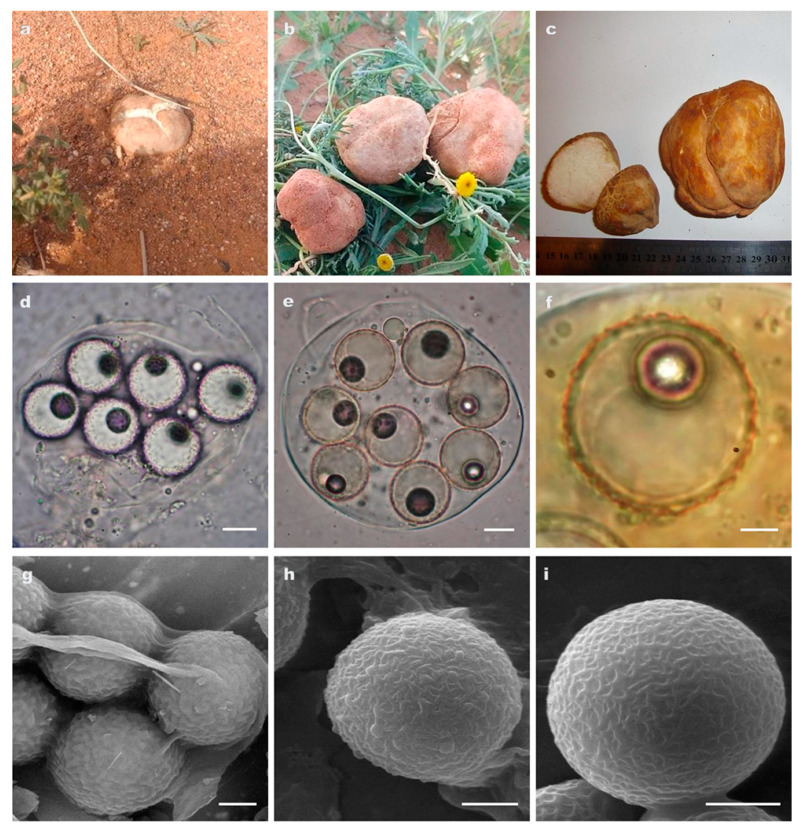
(**a**–**i**) *Tirmania honrubiae*: (**a**,**b**) ascomata in situ; (**c**) ascoma covered with deep depressions and gleba in cross section; (**d**) light microscopy of 6-spored ascus; (**e**) octosporus ascus in Melzer’s reagent showing the very weakly amyloid reaction where the faint blue color does not exceed the ascus wall; (**f**) ornamentation detail of ascospore in Melzer’s reagent under light microscope; (**g**,**h**) scanning electron microscopy of mature ascospores roughened with ridges and low-rounded warts; (**i**) immature young spore ornamented with a well-developed reticulum. Scale bars: 9 μm (**d**,**e**), 8 μm (**f**), and 5 μm (**g**–**i**).

**Description. *Ascomata*** hypogeous, 1–5 cm below soil surface, to partially emergent; subglobose to turbinate with small basal attachment; 2.8–9 × 3–10 cm, 10–197 g fresh weight (Figure 3a–c). *Gleba* solid, fleshy, with yellowish to creamy sterile veins bordering white to pale pink pockets of fertile tissue (Figure 3c)*. Odor* pleasant and strong even after four days; *taste* agreeable.

***Peridium*** 0.46–1.47 mm thick, covered with superficial to deep irregular depressions, orangish yellow to pale brown–orange becoming dark brown with age (Figure 3a–c). ***Asci*** (4–) 6–8 spored; very weakly amyloid (the faint blue color does not exceed asci walls in Melzer’s reagent) to non-amyloid (even from fresh mature samples); subglobose or ellipsoid to pyriform; 63–94 × 48–67 μm, with a short stem 12–24 × 10–15 μm; walls usually not more than 1 μm thick (Figure 3d,e). ***Ascospores*** globose; 15–20 μm diam.; hyaline with a single large guttule and a de Bary bubble at maturity; the wall ± 1 μm thick consists of two layers: outer layer smooth, inner layer roughened with ridges and low rounded warts (0.3–0.8 μm high) which seem to protrude out beyond the outer wall layer at maturity. Young spores appear ornamented with a well-developed reticulum (reticular walls 0.3–0.5 μm thick) which is completely replaced by warts in very mature spores (Figure 3d–i).

**Distribution and Ecology.** ALGERIA, Béchar, Béni Ounif, Oued Namous; Béchar, Abadla, Hammaguir; Oglat Braber; Béni Abbès, Boulaadam; Laghouat, Hassi R’Mel. Hypogeous, solitary, in sandy calcareous, alkaline soil associated with *Helianthemum lippii.* Ascocarps are collected from the beginning of November to the end of April. *Tirmania honrubiae* is locally called «*terfess lahmar*» which means red truffle.

**Collections examined.** ALGERIA, Béchar, Béni Ounif, Oued Namous, March 2012, leg. F.E.-H. Zitouni-Haouar, TM34 (ITS sequence GenBank OM978206, LSU sequence GenBank OM978218), TM35 (ITS sequence GenBank OM978219, LSU sequence GenBank OP871363), AH49191 (ITS sequence GenBank OM985039, LSU sequence GenBank OM985040); ALGERIA, Béchar, Abadla, Hammaguir, April 2012, leg. F.E.-H. Zitouni-Haouar, TM37 (ITS sequence GenBank OM985028), AH49316 (ITS sequence GenBank OM985029), TM39 (ITS sequence GenBank OM985031, LSU sequence GenBank OM978232), AH49195 (ITS sequence GenBank OM985036, LSU sequence GenBank OM985035), March 2013, AH49317 (ITS sequence GenBank OM985032); ALGERIA, Béchar, Abadla, Oglat Braber, April 2015, leg. F.E.-H. Zitouni-Haouar, TM42 (ITS sequence GenBank OM985034, LSU sequence GenBank OM978234), AH49319 (ITS sequence GenBank OM985037, LSU sequence GenBank OM978235), AH49193 (ITS sequence GenBank OM985038), March 2011, TM45 (ITS sequence GenBank OM985933), AH49318 (ITS OM978236); ALGERIA, Béni Abbès, Boulaadam, April 2012, leg. F.E.-H. Zitouni-Haouar, TM47 (ITS sequence GenBank OP871121, LSU sequence GenBank OP871282), TM48 (ITS sequence GenBank OM978238); ALGERIA, Laghouat, Hassi R’Mel, March 2016, leg. F.E.-H. Zitouni-Haouar, TM49 (ITS sequence GenBank OP871283, LSU sequence GenBank OP871360), TM50 (ITS sequence GenBank OP871359, LSU sequence GenBank OP871362).

***Tirmania pinoyi*** (Maire) Malençon

Figure 4

**Figure 4:**
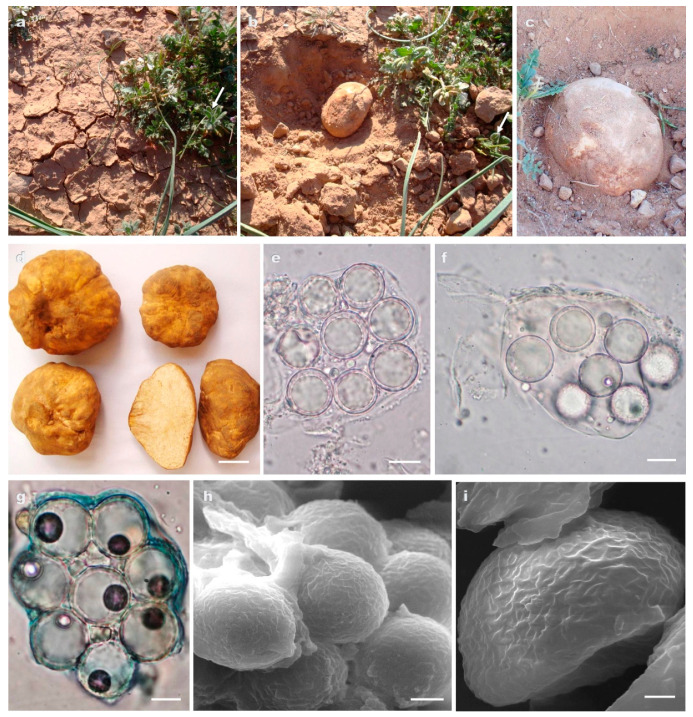
(**a**–**i**) *Tirmania pinoyi*: (**a**) cracks on soil caused by ascoma expansion; (**b**) ascoma after removing ground surface near to the host plant *Helianthemum hirtum* (arrows); (**c**) ascoma in situ; (**d**) ascomata showing peridial surface and gleba in cross section; (**e**,**f**) light microscopy of asci containing ascospores; (**g**) amyloid reaction of octosporus ascus in Melzer’s reagent; (**h**) scanning electron microscopy of mature ascospores roughened with a not well-defined reticulum; (**i**) detail of ascospore surface under SEM. Scale bars: 1.8 cm (**d**), 12 μm (**e**,**f**), 8 μm (**g**), 5 μm (**h**), and 2 μm (**i**).

**Description. *Ascomata*** hypogeous; subglobose to ovoid, or irregularly shaped with flattened basal attachment; 4–7 × 5.5–16.5 cm, 13–480 g fresh weight (Figure 4a–d). *Gleba* solid, fleshy, with white to light yellow or pale pink islets of fertile tissue surrounded by creamy sterile veins (Figure 4d). *Odor* and *taste* pleasant.

***Peridium*** 0.3–1.4 mm thick, superficially to deeply cracked, light brown or yellowish brown becoming slightly darker with age (Figure 4b–d). ***Asci*** amyloid; 6–8 spored at maturity; mostly ellipsoid to ovoid, occasionally pyriform; 50–85 × 30–62 μm, excluding stalk ≤30 µm long; the walls 0.5–1.5 µm thick (Figure 4e–g). ***Ascospores*** globose; 15–20 μm broad.; hyaline with one large guttule and a de Bary bubble which may be lacking at times; the wall up to 1.6 μm thick formed of two layers: outer layer, 0.5–1 μm thick, smooth; inner layer, 0.3–0.6 μm thick, roughened with a not well-defined reticulum (reticular walls showing an irregular polygonal shape) which protrude into the outer wall layer at maturity (Figure 4e–i).

**Distribution and Ecology.** ALGERIA, Tiaret, Bouchouat; Benhamed; Mecheria, El Biodh; Bougarne. Hypogeous, mostly solitary, in sandy loam calcareous, alkaline soil associated with *Helianthemum hirtum, Helianthemum salicifolium*, and *Helianthemum* sp. This species is locally known as «*Belhourech* or *Chehba*» which means truffle with light color. Ascomata are collected from the beginning of February to the middle of June.

**Collections examined.** ALGERIA, Naama, Mecheria, El Biodh, April 2012, leg. F.E.-H. Zitouni-Haouar, AH49194 (ITS sequence GenBank OM845235, LSU sequence GenBank OM845236), TM2 (ITS sequence GenBank OM845238, LSU sequence GenBank OM854800), TM3 (ITS sequence GenBank OM845239, LSU sequence GenBank OM845241); ALGERIA, Naama, Mecheria, Bougarne, March 2013, leg. F.E.-H. Zitouni-Haouar, AH49192 (ITS sequence GenBank OM845245), TM5 (ITS sequence GenBank OM845247, LSU sequence GenBank OM978172), TM6 (ITS sequence GenBank OM978174, LSU sequence GenBank OM978175); ALGERIA, Tiaret, Ksar Chellala, Zmalet El-Amir Abdelkader, Bouchouat, May 2012, leg. F.E.-H. Zitouni-Haouar, TM14 (ITS sequence GenBank OM985023, LSU sequence GenBank OM978180), TM15 (ITS sequence GenBank OM978182, LSU sequence GenBank OM978183), TM16 (ITS sequence GenBank OM978184), TM17 (ITS sequence GenBank OM978185), TM18 (ITS sequence GenBank OM985024, LSU sequence GenBank OM978187), AH49197 (ITS sequence GenBank OM854801, LSU sequence GenBank OM845246); ALGERIA, Tiaret, Hamadia, Rechaiga, Benhamed, February 2009, leg. F.E.-H. Zitouni-Haouar, TM20 (ITS sequence GenBank OM978186), TM21 (ITS sequence GenBank OM985024), TM22 (ITS sequence GenBank OM978189, LSU sequence GenBank OM978191), TM24 (ITS sequence GenBank OM978190, LSU sequence GenBank OM978192), TM25 (ITS sequence GenBank OM985026, LSU sequence GenBank OM978193), TM26 (ITS sequence GenBank OM985027, LSU sequence GenBank OM985030), TM30 (ITS sequence GenBank OM978207).

**Taxonomic key to *Tirmania* species**

1. Peridium orangish yellow to pale brown–orange, spores globose roughened with ridges and low rounded warts……..………………………………… ***T. honrubiae*** (clade 1)
2. Peridium light brown or yellowish brown, spores globose roughened with a not well-defined reticulum………………………………………………………***T. pinoyi*** (clade 2)
3. Peridium off white or yellow to cinnamon, spores smooth exclusively ellipsoid, asci (4–6)–8 spored …………………………………………………………………***T. nivea*** (clade 3)
4. Peridium off white or white yellowish, spores smooth ellipsoid or globose, asci 6–(8–10) spored..……………………………………………………………***T. sahariensis*** (clade 4)

## 4. Discussion

**Figure 5:**
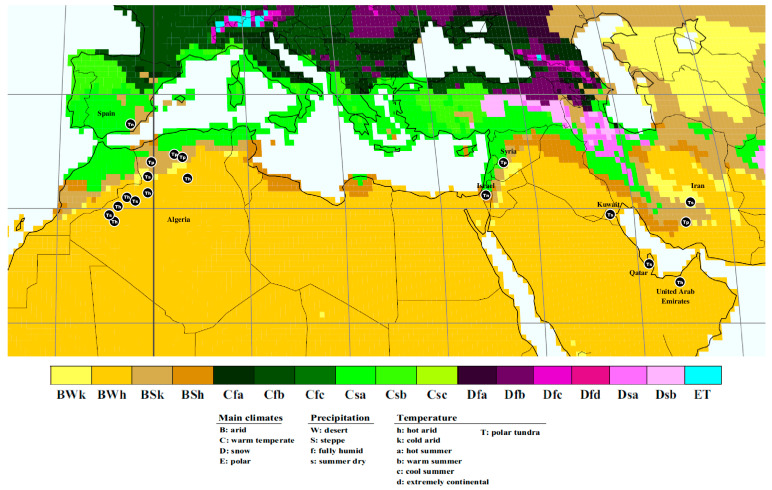
Global bioclimatic distribution of the four *Tirmania* species according to the world map of Köppen–Geiger climate classification updated (Map from Kottek et al. [33]). Tp: *T. pinoyi*, Th: *T. honrubiae*, Tn: *T. nivea*, Ts: *T. sahariensis*.

Phylogenetic and morphological data presented in this study demonstrated that the *Tirmania* genus is even more diverse than previously suspected. The phylogenetic structure of this taxon correlated substantially with morphological features and bioclimatic origin. The main morphological character separating the two major clusters identified in *Tirmania* phylogenies was spore shape. Indeed, the first major group was formed of *T. pinoyi* and *T. honrubiae* with exclusively globose spores, whereas the second one grouped *T. nivea* and *T. sahariensis* showing a mixture of spore shapes with mostly ellipsoid form. However, the species delineation intracluster relates to ascospore morphological characters in addition to the bioclimatic origin. Thus, the major cluster of *Tirmania* species with globose spores includes two genetic lineages on the basis of spore ornamentation (reticulate in *T. pinoyi* and warty in *T. honrubiae*) and according to the bioclimatic niche of the samples, which were collected from a cold arid steppe (BSk) for *T. pinoyi* and a hot arid desert (BWh) for *T. honrubiae* (Figure 1, Table 1) according to the Köppen–Geiger global climate classification [33] (Figure 5). The same situation was observed with the second major cluster of *Tirmania* species with mostly ellipsoid spores which bifurcated into two lineages mainly on the basis of spore form (exclusively ellipsoid in *T. nivea* versus ellipsoid and globose in *T. sahariensis*) and bioclimatic origin. Here again, species delimitation followed the two bioclimatic patterns observed in the first major cluster. Hence, while samples of *T. nivea* were harvested from cold/hot arid steppe (BSk/BSh), those of *T. sahariensis* originated from cold/hot arid desert (BWk/BWh) (Table 1, Figure 1 and Figure 5). This finding suggests that ecological factors and, notably, bioclimatic origin seem to drive the phylogenetic structure of the genus leading to allopatric speciation. Allopatry is generally caused by a geographic barrier that consists of suboptimal environmental conditions for the species in question (e.g., deserts, mountains, or oceans) [52]. The two sets of allopatric siblings species (*T.pinoyi/T.honrubiae, T.nivea/T.sahariensis*) observed in each *Tirmania* major cluster have dissimilar climatic niche envelopes (Arid steppe/Desert climates) and different geographic areas which separate them phylogenetically. Thus, the bioclimatic area range could have a possible role in driving allopatric speciation of the recently discovered *Tirmania* taxon *T. honrubiae* Morte, Bordallo & Ant. Rodr. and the new *Tirmania* species *T. sahariensis* sp. nov. described here, as a response to the natural selective pressures imposed by the hostile conditions of their desert (BWk/BWh) habitats (Figure 1). Natural selection is a central factor affecting speciation and plays an important role in producing phenotypic and genetic diversity within a population. It is the driving force that acts on the existing features of two populations with reference to the ecological differences of their habitats and leads to the evolution of adaptive features [53] (Chethana et al., 2021). Díez et al. [3] were the first to study the intrageneric relationships underlying the phylogeny of the two major desert truffle genera, *Terfezia* and *Tirmania*. They hypothesized that host specialization and edaphic tolerances, especially soil pH (fungus and/or host tolerances), might have played a key role in the speciation of *Terfezia* species. However, these authors could not propose the same hypotheses for *Tirmania* taxa given that they worked on only five specimens. They, therefore, recommended larger surveys to confirm whether *T. nivea* and *T. pinoyi* also have different edaphic tolerances and/or host adaptations. On the other hand, the lack of data on the exact taxonomic identity and phylogenies of the *Helianthemum* species associated with the *Tirmania* samples investigated in the present work calls into question whether the genetic pattern observed in this desert truffle group led to cospeciation with the host plant. In cospeciation, strong congruence (topological similarity) is usually expected between the host and symbiont phylogenies [54]. Further cophylogenetic studies of desert truffle samples with their respective host plants candidates are, therefore, needed to infer the mechanisms of speciation in desert truffles. The phylogenetic diversity examined in this study was strongly supported by morphological evidence and most notably by ascospore and ascus features. Results from the present analyses highlight the importance of spore characters as the most reliable micromorphological features to delimit desert truffle taxa. This was the case also for *Terfezia* and *Picoa* where the species delineation concept was strongly dependent on spore morphological characters, although this diagnostic feature was at times inconspicuous or featureless for delimiting some taxa within these groups. Indeed, morphological similarities recorded in ascospore ornamentation or the absence of this in immature samples were likely the main difficulties that prevailed in the species delimitation of *Terfezia* and *Picoa*, respectively, and which led also to serious taxonomic misidentifications [5,41]. The newly recorded *Tirmania* species from North Africa, *T. honrubiae*, is a further desert truffle species which seems to have been misidentified in the past as *T. pinoyi* due to the great micromorphological similarities between the two taxa. The spore ornamentation which is regarded as the most effective diagnostic tool allowing the discrimination between these two species is hardly detectable with a light microscope and is only perceivable in mature spores under SEM. Alsheikh and Trappe [1] reported that a specimen of *T. pinoyi* collected from an Algerian region (Ain Sefra) characterized by a cold arid desert climate (BWK) was initially deposited in the Patouillard Herbarium (FH) as *Terfezia boudieri*. The morphological and molecular analyses in accordance with bioclimatic data reported here suggest that this specimen may be affiliated to *T. honrubiae* given that it presents globose spores with a BWK bioclimatic origin. The erroneous taxonomic identification of this sample supports our suggestion as the low warts observed on the surface of *T. honrubiae* mature spore strongly resemble those of *T. boudieri*. Although the amyloid reaction of the asci wall is considered as a useful taxonomic identification tool to differentiate *Tirmania* from its closest desert truffles species [11], mature asci even from fresh *T. honrubiae* samples analyzed in the present work were very weakly amyloid to mostly non-amyloid in response to Melzer’s solution. Previous studies reported that the amyloid reaction of the ascus wall, which is considered as the cardinal feature of Pezizaceae, seems to be a symplesiomorphic character which has been lost in several lineages of hypogeous taxa (e.g., in those including *Marcelleina*, *Cazia*, *Terfezia*, and some species of *Pachyphloeus*) [13,14,17] and which seems to be disappearing in *T. honrubiae*. The European Catalogue of hypogeous fungi has also been subject to taxonomic confusion regarding the first report of the genus *Tirmania* from Europe. Indeed, Moreno-Arroyo et al. [21] examined a *Tirmania* material from the Tabernas Desert of Almería (Southern Spain, BSK) and recorded it as *T. pinoyi.* The misdiagnosis was revealed later when Moreno et al. [22] studied the same material and reported that it displayed ellipsoid spores (never spherical) typical of *T. nivea*. The molecular and phylogenetic analysis presented here and performed on the same *Tirmania* material from the Tabernas Desert (MA Fungi 37352) (Figure 1, Table 1) analyzed in the two previous studies supported the emendation made by Moreno et al. [22] regarding the abolition of *T. pinoyi* from the Spanish hypogeous fungal flora and the record of *T. nivea* as the unique representative species of the genus *Tirmania* in the European mycoflora. The *T. nivea* combination was originally proposed by Trappe [11] to represent the *Tirmania* taxon with broadly ellipsoid, smooth spores. The phylogenetic inferences based on the rDNA sequence dataset and the mixture of globose and ellipsoid spores as well as the eight- to ten-spored asci observed in specimens of the *T. nivea* sister lineage (Figure 1) provide strong evidence for the erection of the new *Tirmania* species, *T. sahariensis* sp. nov.

## 5. Conclusions

This work addressed the intrageneric relationships among *Tirmania* species using morphological and phylogenetic analysis and considering bioclimatic information. Our analyses provide the first insights into the influence of climatic range area on the evolution of *Tirmania* species. The genetic structure of this desert truffle group seems to be related to climatic niche divergence leading to allopatric speciation. We confirmed the existence of four distinct genetic lineages within this group corresponding to four morphological species with a new species and a new record. Further phylogenomic analysis from whole genomic sequencing of *Tirmania* strains are needed in order to refine phylogenies and to fully understand the observed genetic pattern. Phylogenies presented in this study have led to a taxonomic update of *Tirmania* and provided essential phylogenetic background for the conservation and management of these highly valued edible fungi.

## Data Availability

All sequences generated in this study were submitted to GenBank.

## References

[1] Alsheikh A.M., Trappe J.M. (1983). Desert truffles: The genus *Tirmania*. Trans. Br. Mycol. Soc..

[2] Alsheikh A.M. (1994). Taxonomy and Mycorrhizal Ecology of the Desert Truffles in the Genus *Terfezia*. Ph.D. Thesis.

[3] Díez J., Manjón J.L., Martin F. (2002). Molecular phylogeny of the mycorrhizal desert truffles (*Terfezia* and *Tirmania*), host specificity and edaphic tolerance. Mycologia.

[4] Zitouni-Haouar F.E.-H., Fortas Z., Chevalier G. (2014). Morphological characterization of mycorrhizae formed between three *Terfezia* species (desert truffles) and several Cistaceae and Aleppo pine. Mycorrhiza.

[5] Zitouni-Haouar F.E.-H., Carlavilla J.R., Moreno G., Manjon J.L., Fortas Z. (2018). Genetic diversity of the genus *Terfezia* (Pezizaceae, Pezizales): New species and new record from North Africa. Phytotaxa.

[6] Morte A., Gutiérrez A., Navarro-Ródenas A. (2020). Advances in desert truffle mycorrhization and cultivation. Mushrooms, Humans and Nature in a Changing World.

[7] Morte A., Kagan-Zur V., Navarro-Ródenas A., Sitrit Y. (2021). Cultivation of desert truffles—A crop suitable for arid and semi-arid zones. Agronomy.

[8] Chatin A. (1891). Contribution à l′histoire naturelle de la truffe. Bull. Société Bot. Fr..

[9] Chatin A. (1892). La Truffe.

[10] Patouillard N. (1892). Énumération des champignons observés en Tunisie. Exploration Scientifique de la Tunisie.

[11] Trappe J.M. (1971). A synopsis of the Carbomycetaceae and Terfeziaceae (Tuberales). Trans. Br. Mycol. Soc..

[12] Trappe J.M. (1979). The orders, families and genera of hypogeous ascomycotina (truffles and their relatives). Mycotaxon.

[13] Hansen K., Læssøe T., Pfister D.H. (2001). Phylogenetics of the Pezizaceae, with an emphasis on *Peziza*. Mycologia.

[14] Hansen K., Lobuglio K.F., Pfister D.H. (2005). Evolutionary relationships of the cup-fungus genus *Peziza* and Pezizaceae inferred from multiple nuclear genes: RPB2, β-tubulin, and LSU rDNA. Mol. Phylogenet. Evol..

[15] Ferdman Y., Aviram S., Roth-Bejerano N., Trappe J.M., Kagan-Zur V. (2005). Phylogenetic studies of *Terfezia pfeilii* and *Choiromyces echinulatus* (Pezizales) support new genera for southern African truffles: *Kalaharituber* and *Eremiomyces*. Mycol. Res..

[16] Tedersoo L., Hansen K., Perry B.A., Kjøller R. (2006). Molecular and morphological diversity of pezizalean ectomycorrhiza. New Phytol..

[17] Læssøe T., Hansen K. (2007). Truffle trouble: What happened to the Tuberales?. Mycol. Res..

[18] Kovács G.M., Trappe J.M., Kagan-Zur V., Roth-Bejerano N., Sitrit Y., Morte A. (2014). Nomenclatural History and Genealogies of Desert Truffles. Soil Biology.

[19] Maire R. (1906). Notes mycologiques. Ann. Mycol..

[20] Malençon G. (1973). Champignons hypogés du Nord de, l’Afrique–I. Ascomycetes. Persoonia.

[21] Moreno-Arroyo B., Gomez J., Calonge F.D. (1997). Aportaciones a la micoflora hipogea Ibérica. Boletín Soc. Micol. Madr..

[22] Moreno G., Díez J., Manjon J.L. (2000). *Picoa lefebvrei* and *Tirmania nivea*, two rare hypogeous fungi from Spain. Mycol. Res..

[23] Kovács G.M., Balazs T.K., Calonge F.D., Martín M.P. (2011). The diversity of *Terfezia* desert truffles: New species and a highly variable species complex with intrasporocarpic nrDNA ITS heterogeneity. Mycologia.

[24] Bordallo J.J., Rodríguez A., Honrubia M., Morte A. (2012). *Terfezia canariensis* sp. nov. una nueva especie de trufa encontrada en las Islas Canarias. Cantarela.

[25] Bordallo J.J., Rodríguez A., Muñoz-Mohedano J.M., Suz L.M., Honrubia M., Morte A. (2013). Five new *Terfezia* species from the Iberian Peninsula. Mycotaxon.

[26] Bordallo J.J., Rodríguez A., Kaounas V., Camello F., Honrubia M., Morte A. (2015). Two new *Terfezia* species from Southern Europe. Phytotaxa.

[27] Bordallo J.J., Rodriguez A., Santos-Silva C., Louro R., Muñoz-Mohedano J.M., Morte A. (2018). *Terfezia lusitanica*, a new mycorrhizal species associated to *Tuberaria guttata* (Cistaceae). Phytotaxa.

[28] Bordallo J.J., Rodriguez A., Morte A. (2018). *Terfezia morenoi.* Fungal Planet description sheets. Persoonia.

[29] Moreno G., Manjón J.L., Alvarado P. (2019). A new *Terfezia* from Spain. Boletín Soc. Micol. Madr..

[30] Rodríguez A., Navarro-Ródenas A., Morte A., Cabero J., Luque D. (2019). *Terfezia dunensis*. Fungal Planet description sheets. Persoonia.

[31] Louro R., Nobre T., Santos-Silva C. (2020). *Terfezia solaris-libera* sp. nov., a new mycorrhizal species within the spiny-spored lineages. J. Mycol. Mycol. Sci..

[32] Morte A., Bordallo J.J., Rodriguez A. (2018). *Tirmania honrubiae*. Fungal Planet description sheets. Persoonia.

[33] Kottek M., Grieser J., Beck C., Rudolf B., Rubel F. (2006). World map of the Köppen-Geiger climate classification updated. Meteorol. Z..

[34] Langeron K. (1952). Précis de Mycologie.

[35] Moreno G., Altés A., Ochoa C., Wright J.E. (1995). Contribution to the study of the family *Tulostomataceae* in Baja California, Mexico. Mycologia.

[36] Gardes M., Bruns T.D. (1993). ITS primers with enhanced specificity for Basidiomycetes-application to the identification of mycorrhizae and rusts. Mol. Ecol..

[37] Bidartondo M.I., Burghardt B., Gebauer G., Bruns T.D., Read D.J. (2004). Changing partners in the dark: Isotopic and molecular evidence of ectomycorrhizal liaisons between forest orchids and trees. Proc. R. Soc. Lond. B.

[38] White T.J., Bruns T.D., Lee S., Taylor J.W., Innis M.A., Gelfand D.H., Sninsky J., White T.J. (1990). Amplification and direct sequencing of fungal ribosomal RNA genes for phylogenetics. PCR Protocols: A Guide to Methods and Applications.

[39] Vilgalys R., Hester M. (1990). Rapid genetic identification and mapping of enzymatically amplified ribosomal DNA from several *Cryptococcus* species. J. Bacteriol..

[40] Cubeta M.A., Echandi E., Abernethy T., Vilgalys R. (1991). Characterization of anastomosis groups of binucleate *Rhizoctonia* species using restriction analysis of an amplified ribosomal RNA gene. Phytopathology.

[41] Zitouni-Haouar F.E.-H., Alvarado P., Sbissi I., Boudabous A., Fortas Z., Moreno G., Manjón J.L., Gtari M. (2015). Contrasted genetic diversity, relevance of climate and host plants, and comments on the taxonomic problems of the genus *Picoa* (Pyronemataceae, Pezizales). PLoS ONE.

[42] Murray M.G., Thompson W.F. (1980). Rapid isolation of high molecular weight plant DNA. Nucleic Acids Res..

[43] Mullis K.B., Faloona F.A. (1987). Specific synthesis of DNA *in vitro* via a polymerase-catalyzed chain reaction. Methods Enzymol..

[44] Altschul S.F., Madden T.L., Schaffer A.A., Zhang J.H., Zhang Z., Miller W., Lipman D.J. (1997). Gapped BLAST and PSI-BLAST: A new generation of protein database search programs. Nucleic Acids Res..

[45] Edgar R.C. (2004). MUSCLE: Multiple sequence alignment with high accuracy and high throughput. Nucleic Acids Res..

[46] Tamura K., Stecher G., Kumar S. (2021). MEGA11: Molecular Evolutionary Genetics Analysis Version 11. Mol. Biol. Evol..

[47] Jamali S., Banihashemi Z. (2012). Hosts and Distribution of Desert Truffles in Iran, based on morphological and molecular criteria. J. Agric. Sci. Technol..

[48] Osmundson T.W., Robert V.A., Schoch C.L., Baker L.J., Smith A., Robich G., Mizzan L., Garbelotto M.M. (2013). Filling gaps in biodiversity knowledge for macrofungi: Contributions and assessment of an herbarium collection DNA barcode sequencing project. PLoS ONE.

[49] Miller M.A., Pfeiffer W., Schwartz T. Creating the CIPRES Science Gateway for inference of large phylogenetic trees. Proceedings of the 2010 Gateway Computing Environments Workshop (GCE).

[50] Posada D. (2008). jModelTest: Phylogenetic model averaging. Mol. Biol. Evol..

[51] Ronquist F., Teslenko M., Van der Mark P., Ayres D.L., Darling A., Hohna S., Larget B., Liu L., Suchard M.A., Huelsenbeck J.P. (2012). MrBayes 3.2: Efficient Bayesian phylogenetic inference and model choice across a large model space. Syst. Biol..

[52] Wiens J.J., Graham C.H. (2005). Niche conservatism: Integrating evolution, ecology, and conservation biology. Annu. Rev. Ecol. Evol. Syst..

[53] Chethana K.T., Manawasinghe I.S., Hurdeal V.G., Bhunjun C.S., Appadoo M.A., Gentekaki E., Raspé O., Promputtha I., Hyde K.D. (2021). What are fungal species and how to delineate them?. Fungal Divers..

[54] Giraud T., Refrégier G., Le Gac M., de Vienne D.M., Hood M.E. (2008). Speciation in fungi. Fungal Genet. Biol..

